# Enhanced Microbial Diversity Attained Under Short Retention and High Organic Loading Conditions Promotes High Volatile Fatty Acid Production Efficiency

**DOI:** 10.3390/molecules31010132

**Published:** 2025-12-30

**Authors:** Claudia Chao-Reyes, Rudolphus Antonius Timmers, Ahmed Mahdy, Silvia Greses, Cristina González-Fernández

**Affiliations:** 1 Department of Chemical Engineering and Environmental Technology, School of Industrial Engineering, University of Valladolid, Dr. Mergelina, 47011 Valladolid, Spain; claudia.chao@uva.es; 2 Institute of Sustainable Processes, Dr. Mergelina, 47011 Valladolid, Spain; 3 CARTIF Technology Centre-Area of Circular Economy, Av. Francisco Vallés, 4, 47151 Boecillo, Spain; rudant@cartif.es; 4 Department of Agricultural Microbiology, Faculty of Agriculture, Zagazig University, Zagazig 44511, Egypt; aamahdi@zu.edu.eg; 5 CALAGUA–Unidad Mixta UV-UPV, Department of Chemical Engineering, Universitat de València, Av. de la Universitat s/n, 46100 Burjassot, Spain; silvia.greses@uv.es

**Keywords:** anaerobic fermentation, volatile fatty acids, agro-industrial waste, operational parameters

## Abstract

The optimization of volatile fatty acid (VFA) production from complex wastes under anaerobic conditions remains constrained in terms of productivity by the common use of long hydraulic retention times (HRTs, 20–30 days). Extended HRTs can limit process productivity by reducing substrate turnover and reactor throughput, while promoting further conversion of VFAs into methane and other end products. Despite its importance, the combined influence of pH and HRT on VFA yields and process optimization has not been comprehensively evaluated. This study investigates the effects of pH and short HRT on VFA production, microbial community structure, and hydrolysis and acidification efficiency in continuous stirred-tank reactors (CSTRs) fed with carbohydrate-rich feedstock (carrot residue pulp). Operating at an HRT of 11 days and an organic loading rate (OLR) of 4.4 g COD·L^−1^·d^−1^ at 25 °C under pH 5.1 resulted in a VFA bioconversion efficiency of ~45% and an acidification efficiency of 84%, without compromising VFA profile or productivity compared to reactors operated at 14 days HRT and 3.3 g COD·L^−1^·d^−1^. The shorter HRT and higher OLR enhanced hydrolysis efficiency (60%) and promoted greater microbial diversity, supporting robust hydrolytic activity and stable production dominated by acetic and butyric acids. These findings challenge the conventional assumption that longer retention times inherently improve process stability and demonstrate that operational conditions might improve reactor space–time yield in VFA-oriented fermentations.

## 1. Introduction

Unsustainable food production systems are currently a problem caused by high food consumption driven by overpopulation worldwide. One of the main issues associated with overproduction is the vast amount of food waste generated. According to the United Nations Environment Programme (UNEP), 17% of the world’s total food production was discarded in 2019. This phenomenon has a negative environmental impact, as the decomposition of discarded food generates between 8% and 10% of global greenhouse gas emissions [1].

The increasing generation of waste highlights a pressing need—and a valuable opportunity—to adopt circular economic practices that transform waste into resources, reduce environmental impact, and drive sustainable growth. In order to valorize food waste, one relatively straightforward technology is the use of anaerobic digestion (AD), which converts waste into a valuable resource for the generation of new products, thus contributing to a circular economy. AD conventionally leads to biogas production for energy purposes. However, over the last decade, AD has been modified to produce valuable chemicals that can replace some petroleum derivates. One of the products that can be obtained via modified AD is volatile fatty acids (VFAs), which are widely used as chemical platforms for the production of biofuels and other bioproducts (microbial oils and polyhydroxyalkanoates) [2,3]. VFAs can be produced by inhibiting the final stage of AD—methanogenesis. To achieve their accumulation in the system, it is crucial to reduce the population or inhibit the activity of methanogenic archaea, as they consume acetate for biogas production purposes [3]. Instead, acidogenic bacteria must be promoted to maximize soluble organic matter conversion into VFAs. For instance, previous studies determined that *Ruminococcus* and *Bifidobacteria* were key bacteria to degrade sugar-rich residues into even-chain VFAs, namely, acetic and butyric acid [4].

The substrate and operational parameters of the process, such as hydraulic retention time (HRT), organic loading rate (OLR), pH and temperature, are key to inhibiting the activity of methanogenic archaea while promoting that of acidogens, ultimately leading to the accumulation of VFAs in the system. Bolzonella et al. [5] and Gonçalves et al. [6] reported that pH values between 5.5 and 6.5 favor VFA production over biogas. Higher pHs favor the activity of methanogens, which thrive at around pH 7.0–7.5, while lower pHs mediate the accumulation of primary metabolites (ethanol and lactic acid) [7].

High OLRs and short HRTs accelerate acidogenesis, causing rapid VFA accumulation and pH decline that inhibit slow-growing methanogens. When methanogens are suppressed—either by high substrate loading or insufficient retention time—more carbon flows toward VFA formation, increasing VFA production efficiency up to an optimal threshold. However, an excessively high OLR or a very short HRT can trigger severe acidification and biomass washout, ultimately reducing the efficiency and stability of VFA production. Therefore, another strategy to overcome methanogen activity is the use of a short HRT because their growth rate is lower than that of acidogenic bacteria [3]. Nevertheless, operating with an HRT below a critical threshold may compromise the growth of hydrolytic and acidogenic bacteria as well. However, when selecting an appropriate HRT, an important factor to consider is the composition of the substrate. In this regard, Swiatkiewicz et al. [8] demonstrated that, when working with complex particulate substrates, sufficiently long HRTs are necessary to enable the hydrolysis of complex organic matter. To prevent the proliferation of methanogenic archaea under these conditions, Gonçalvez et al. [6] indicated that combining long HRTs with acidic pH conditions can be an effective strategy to enhance VFA production and inhibit methanogenesis. Indeed, most researchers use long HRTs (20–30 days) as the hydrolysis of complex wastes requires an extended period [9,10]. However, long HRTs affect productivity and can lead to lower process efficiency, as the volumetric rate of VFA production decreases due to slower substrate turnover and reduced reactor throughput. Additionally, extended retention times may favor further conversion of VFAs into methane or other end products, thereby reducing the accumulation of the desired intermediates. Currently, the literature lacks a comprehensive evaluation of how pH and HRT jointly affect VFA yields and process optimization in carbohydrate-rich anaerobic fermentations, hindering the identification of time-efficient operational strategies. To further clarify which of the operational parameters exerts greater influence concerning the optimization of VFA production, without compromising concentrations, yields or productivity, this investigation evaluated the influence of pH and short HRT in an anaerobic fermentation process using carbohydrate-rich substrates. Special attention was given to the microbial communities developed to understand how shifts in community composition and diversity correlate with process performance, hydrolysis efficiency, and VFA profiles under different operational conditions.

## 2. Results and Discussion

### 2.1. Fermentation Robustness upon Operational Changes When Targeting at VFAs Production

Table 1 shows the results obtained during the stable phase of the anaerobic fermentation process (third retention time onwards). The average VFA concentration was 15.9 ± 1.4 g/L for R1 operated at pH 5.1, while a slightly lower concentration (13.7 ± 2.4 g/L) was attained in R2 operated at pH 6. Taking into consideration the COD equivalents of each of the VFAs, the bioconversion yields reached 53.6 ± 5.4% and 45.4 ± 8.5% in R1 and R2, respectively. The same trend was also determined for hydrolysis and acidification efficiency. Atasoy and Cetecioglu [11] reported similar results for two anaerobic fermentation processes operated under acidic (pH = 5.0) and neutral (pH = 7.0) conditions, obtaining the highest VFA production at the lowest pH (pH = 5.0). These results demonstrated that acidic pH conditions can enhance hydrolysis performance, contrary to the conventional assumption that an alkaline pH is more favorable in this stage. In the present investigation, both pHs supported similar VFA concentration and bioconversions (53.6 ± 5.4 and 45.4 ± 8.5% for R1 and R2, respectively, Table 1). Interestingly, R1 achieved the highest yield despite operating outside of the optimum pH range (5.5–6.0) reported for VFA production from carbohydrate-rich substrates [12]. This could be related to the HRT implemented as the optimal pH range was previously identified for a short HRT (9–10 days [13]), whereas R1 and R2 were operated at a longer HRT (14 days). These findings are consistent with the observations of Gonçalvez et al. [6], who highlighted that the combination of extended HRTs with acidic pH conditions can stimulate acidogenic bacterial activity while simultaneously suppressing methanogenic populations. Operating at pH values below the range also entails a reduction in process costs because less sodium hydroxide is required to control pH throughout the experiment. According to Garcia-Peña et al. [14], anaerobic degradation of agricultural wastes might lead to a significant pH drop (down to 4) that inhibits the bioprocess of organic matter degradation. Although pH 4 should not be reached, the results attained at pH 5 in R1 would result in economic benefits and a less complex system compared to pH 6 (R2) but with similar results for VFA concentration and bioconversion. A pH of 5 can be more readily achieved due to the self-acidification of food waste. In fact, this involves a dual benefit as archaea were also inhibited and no biogas production was detected. This is of particular relevance for maintaining a strictly fermentative environment, ensuring that the produced intermediates (e.g., VFAs) are not further converted to methane, thereby enhancing substrate conversion efficiency and process selectivity.

A similar trend was determined for acidification efficiency. In the case of the hydrolysis stage (based on VS removal), slightly higher efficiencies (53.2 ± 1.3%) were recorded for R1 at pH 5. This could be explained by the fact that a pH of 5 provides a more favorable environment for hydrolytic enzyme activity during food waste anaerobic fermentation, as the enzymes exhibit optimal stability and catalytic efficiency under mildly acidic conditions [15]. In contrast, at pH 6.5, reduced proton availability and altered enzyme ionization states can diminish substrate binding and overall reaction rates, decreasing hydrolysis efficiency. This finding confirmed that a higher process pH results in lower bioconversion efficiency into VFAs, as hydrolytic activity is compromised. It should be stressed that hydrolytic inefficiencies are commonly regarded as the most likely cause of low bioconversion of complex organic matter. However, acidogenic inhibition has also been reported at high OLRs rather than as a result of traditional hydrolytic barriers. In the present investigation, acidification yields reached comparable values in both reactors, R1 and R2, regardless of the process pH, which corroborates the finding that the bioconversion increase in R1 was mainly related to high hydrolytic activity at pH 5.

**Figure 1:**
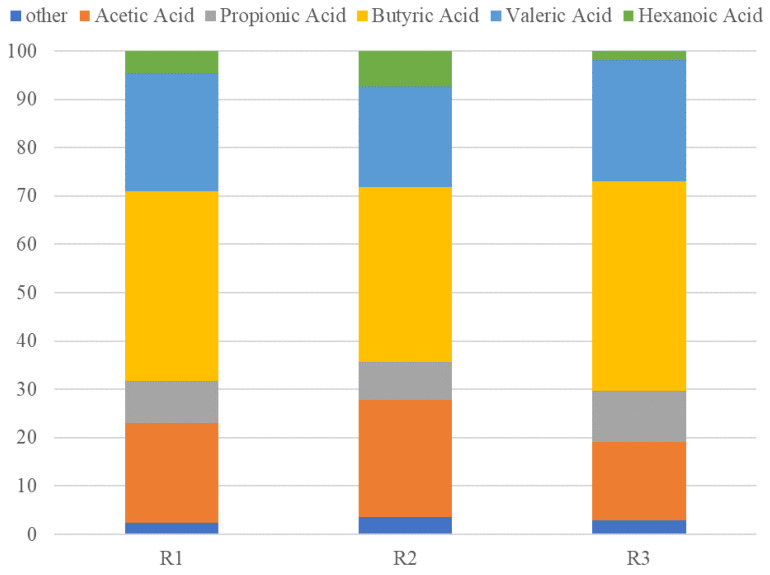
VFA distribution profiles determined in each reactor.

Nevertheless, the operational pH did not result in changes in the VFA distribution profile (Figure 1). According to the obtained data, HAc and HBu were the predominant components in the VFA distribution profile for all reactors (Figure 1). This similarity may be attributed to the established operating conditions and the residue used as feedstock, as Gonçalvez et al. [6] reported that anaerobic fermentation of carbohydrate-rich substrates under acidic conditions favors the production of those VFAs (HAc and HBu). More importantly, high selectivity for even-chain VFAs can be concluded as the sum of C2 (acetic acid) and C4-VFAs (butyric acid) accounted for approx. 65% of the total VFA pool. This is of particular importance as product selectivity simplifies the separation and purification steps. This directly lowers downstream processing costs and improves overall process efficiency and yield.

Based on the results attained when operating in R1 at pH 5 (the highest VFA concentration), the bioprocess was evaluated at a higher OLR (4.1 g COD/Ld) and shorter HRT (11 days). As can be seen in Table 1, those changes provoked a VFA concentration decrease (11.6 ± 1.4 g/L) compared to R1, corresponding to a bioconversion efficiency of 43.7 ± 6.4%. Regarding acidification and hydrolysis efficiency, values of 83.7 ± 10.3% and 60.1 ± 7.2% were attained for this reactor operating at 11 d of HRT, which were similar values to those reached in R1. According to these results, acidification efficiency was not affected by HRT nor by pH, but hydrolysis was affected by HRT (and OLR), showing a slight increase. When OLR increases for a carbohydrate-rich feedstock, more readily degradable substrate becomes available for hydrolytic and fermentative bacteria, stimulating their growth and enzyme production. This leads to the faster conversion of polysaccharides into soluble sugars and VFAs, thus improving hydrolysis efficiency. However, this effect only holds up to a certain point—beyond the optimal OLR, acid accumulation and pH drops can inhibit further hydrolysis. As hydrolysis efficiency was lower at lower OLRs, it can be hypothesized that the microbial system was under substrate-limited conditions, resulting in reduced microbial activity. Indeed, productivity was also not affected by a decrease in HRT (1.05 ± 0.1 g/Ld, Table 1). In terms of VFA concentration, the values obtained herein were higher than those reported in the literature when operating at a similar HRT with the same reactor configuration. For instance, Bolaji and Dionisi [16] reported VFA concentrations of 9.1 g COD-VFAs/L when operating with a HRT of 10 days with an acidic pH at 35 °C. This value was remarkably lower than the VFA concentration attained in R3 when considering the COD contribution of the VFAs—the reactor displayed 19.6 ± 1.9 g COD-VFAs/L (Table 1). Despite operating under acidic conditions and using similar HRTs, their concentrations were lower than those obtained in this study—likely due to the use of a higher temperature (35 °C), which may have stimulated methanogenesis and thus reduced VFA concentration levels. Even though conventional anaerobic digestion is conventionally run at 35 °C, research conducted for VFA production has demonstrated that 25 °C seems to be more beneficial [17].

Regarding the VFA distribution profile (Figure 1), a high selectivity of HAc and HBu was also evidenced. In this case, C2+C4-VFAs accounted for 60% of the VFA concentration pool. It could be concluded that the decrease in HRT (and concomitant increase in OLR) did not affect the distribution profile. Despite the changes, the microbial community was shown to be able to cope with the harsh implemented conditions by supporting a reproducible output at the chemical level.

### 2.2. Microbial Community Changes upon Implemented Operational Conditions

To analyze the microbial community present in the inoculum and in the microbiomes developed during the steady state of the reactor’s performance for VFA production, biodiversity was assessed in terms of richness and evenness. The inoculum exhibited high biodiversity, as reflected by the number of operational taxonomic units (OTUs) and the Shannon index (Table 2). This was expected as the inoculum was collected using a conventional anaerobic digester operating at a low OLR and long HRT, thereby enabling the presence of fast and slow growers. When considering the microbiomes retrieved from the fermenters targeting VFA production, both parameters decreased, in terms of diversity and evenness, due to the imposed conditions, which favored the growth of bacteria able to thrive in harsh conditions. A shorter HRT might increase microbial diversity because it adds disturbance, increases the inflow of resources, and prevents slow-growing taxa from dominating, thereby allowing more fast-growing or niche-adapted microbes to coexist. This trend has been previously reported when conventional anaerobic microbiomes are subjected to organic matter degradation for VFA production under harsh conditions, such as low temperature and pH [6,18].

The lower OTU count registered for R1 and R2 represented the loss of richness whereas the decrease in Shannon index implied that the microbial community became dominated by a few species rather than being evenly distributed. Operational conditions (e.g., higher OLR, pH changes, VFA accumulation) resulted in a smaller number of specialized microbes that thrive under those conditions, while more sensitive or less competitive species declined (when compared to the inoculum). The decrease in Shannon index of microbiomes subjected to conditions different to common AD has been shown to follow the same pattern, in which the index is much higher for the inoculum used to seed the reactors [19].

**Figure 2:**
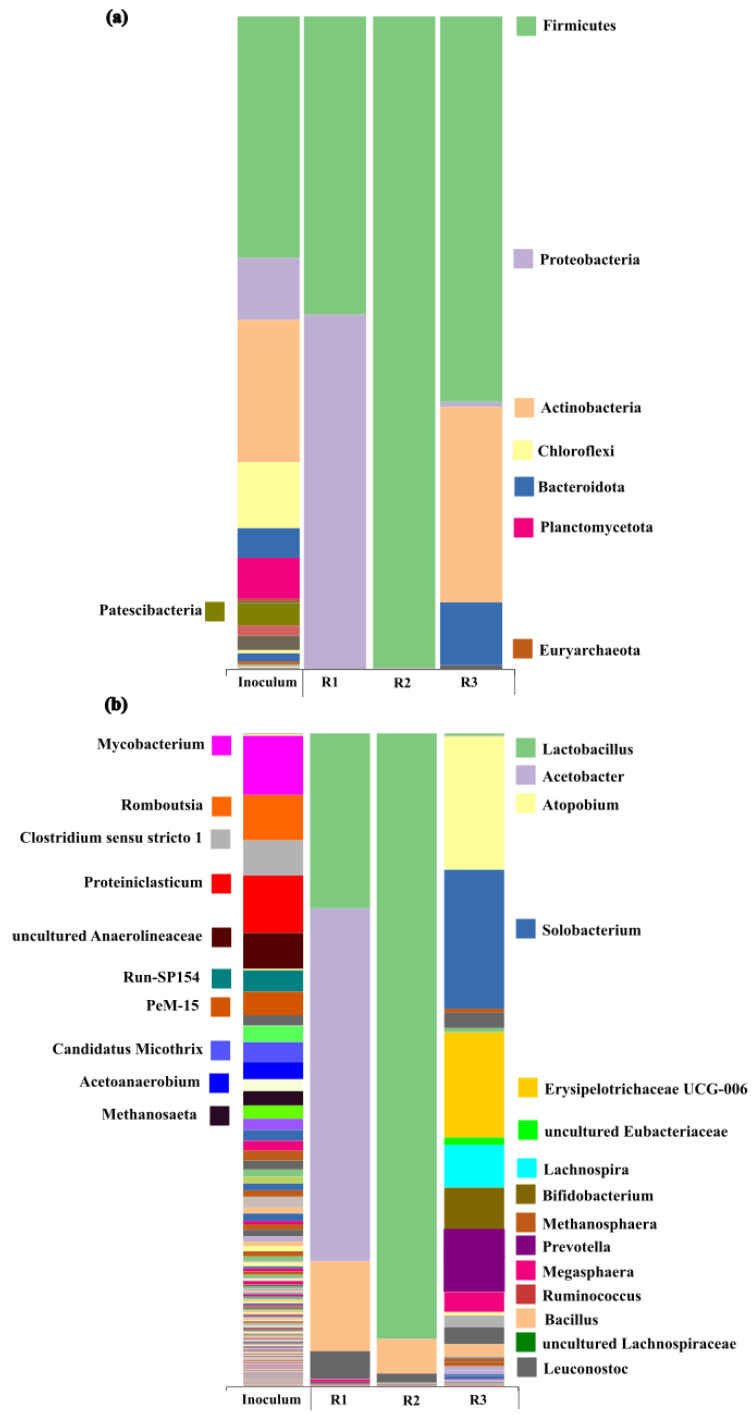
Relative abundance of bacteria and archaea identified in the inoculum and the reactors at (**a**) phylum and (**b**) genus level.

Figure 2 shows the microbial abundance of phyla (a) and genera (b) for the inoculum used in the fermenters and the microbiomes developed during the operation of each reactor. In the case of the inoculum, a heterogeneous phylum distribution was observed, mainly composed of Firmicutes (36.9%), Actinobacteriota (21.8%), Chloroflexi (10.0%), Proteobacteria (9.5%) and Bacteroidota (4.5%). These are typical communities of mesophilic anaerobic digestion (35 °C) when targeting biogas production [20,21]. Firmicutes and Chloroflexi are associated with carbohydrate degradation, Proteobacteria with protein and cellulose degradation [15], whereas Actinobacteria and Bacteroidota are responsible for breaking down complex macromolecules such as cellulose [6,22]. Planctomycetota (6.2%) and Patescibacteria (3.4%) were also identified in smaller abundance. Members of Planctomycetota are commonly found in soils, freshwater and marine ecosystems [23]. Their presence in AD processes, even at lower abundance, has been reported by Campanaro et al. [24] and Klimek et al. [25], who associated them with the degradation of complex sugars as they encode various carbohydrate-active enzymes. In turn, the phylum Patescibacteria, characterized by a reduced genome and highly limited metabolic capacities, has been detected in AD in symbiosis with other bacteria such as Firmicutes [26]. Overall, the microbiome of the inoculum presented a wide range of microbial activities related to the different phyla determined.

As mentioned above and as can be observed in Figure 2, when compared to the inoculum, the bacterial community decreased in both diversity and evenness during anaerobic fermentation. Once the steady state was reached in R1, the phylum distribution was dominated by Proteobacteria (54.2%) and Firmicutes (45.6%). A more drastic change was determined in R2, in which Firmicutes were predominant (99.8%). This shift was due to the operating conditions, which promoted the prevalence of microbial communities involved in anaerobic fermentation for VFA production, instead of those more related to anaerobic digestion. The pH difference between the two reactors influenced the microbial community, as the increase from 5.1 to 6.0 resulted in the disappearance of Proteobacteria. This feature was unexpected as acidic environments normally favor acid-tolerant groups like Actinobacteria, which outcompete Proteobacteria [27]. Proteobacteria disappearing at pH 6 (R2) was therefore not likely due to pH itself but rather attributed to changes associated with nutrient availability or microbial competitors. Firmicutes also prevailed within the microbiome developed at pH 5 with a HRT of 11 d (R3). However, instead of Proteobacteria (as observed in R2), Firmicutes were thriving along with Actinobacteria and Bacteroidetes. Previous studies have also shown a predominance of Actinobacteria at longer HRTs [28]. Members of both Bacteroidetes and Actinobacteria are involved in the degradation of complex carbohydrates and the subsequent production of acetic and butyric acids [3]. Overall, these phyla are essential for hydrolysis and acidogenesis, as they degrade carbohydrates and proteins to produce VFAs [29]. Firmicutes have been claimed to be closely associated with VFA production from carbohydrate-rich substrates, which also explains their high abundance in this study and is consistent with the high hydrolysis yields obtained (50–60% VS removal, Table 1). Also relevant was the fact that the inoculum exhibited the presence of Euryarchaeota (archaea), which fully disappeared upon the operational conditions selected to produce VFAs (Figure 2a). This finding demonstrates that the operational conditions were properly selected to limit methanogen growth, evidencing the successful strategy of combining HRTs with low pH and temperature, without compromising VFA production.

According to genus-level classification, the inoculum exhibited high biodiversity, with the most abundant genera being *Romboutsia* (6.9%), *Mycobacterium* (9.0%), *Proteiniclasticum* (8.7%), *Clostridium sensu stricto 1* (5.3%) and uncultured Anaerolineaceae (5.4%). To a lesser extent, *Run-SP154* (3.2%), *Acetoanaerobium* (2.6%), *Candidatus_Microthrix* (2.3%), *PeM15* (3.5%), and *Methanosaeta* (2.1%) were also observed. Many bacteria belonging to these genera are involved in different stages of anaerobic degradation. For instance, *Romboutsia* and *Clostridium sensu stricto 1* are abundant in processes targeted at VFAs/hydrogen production [30]. Similarly, *Proteiniclasticum* has been previously reported to utilize carbohydrate and protein sources to produce HAc and HPro [31]. On the other hand, the presence of *Methanosaeta* may indicate that acetoclastic methanogenesis was the predominant pathway for methane production when the inoculum was working in conventional digesters.

In the case of microbiomes developed in the fermenters for VFA production, the shift at genera level was also remarkable. Within the Proteobacteria phylum retrieved in R1 operated at pH = 5.1 and HRT 14 d, the genus *Acetobacter* (54.0%) was predominant, whereas within the Firmicutes, the genera *Lactobacillus* (26.7%), *Bacillus* (13.7%) and *Leuconostoc* (4.2%) were identified. Strikingly, R2 operated at pH = 6.0 and HRT 14 d exhibited almost full predominance of *Lactobacillus* (92.7%). The presence of *Lactobacillus* and *Leuconostoc* is associated with lactic acid production and, to a lesser extent, HAc [32,33]. It is therefore possible that lactic acid was produced and subsequently used as an electron donor in the reverse β-oxidation process, thereby elongating HPro to HVal [6,34] as reflected in the high HVal concentrations obtained in both reactors (Figure 1). It should be noted that *Lactobacillus* has also been associated with HBu production, whereas *Bacillus* is directly related to carbohydrate degradation and the formation of VFAs such as HAc and HBu [28]. Their high abundance explains the high concentrations of both VFAs (HAc and HBu).

Interestingly, the microbial abundance at the genera level significantly increased when the HRT was decreased to 11 d combined with pH 5.1 (R3) (Figure 2b). The increase in genera was linked to the higher OLR implemented in R3 to decrease the HRT. In this case, the dominant Firmicutes genera were *Solobacterium* (21.2%), Erysipelotrichaceae UCG-006 (16.1%) and *Lachnospira* (6.5%). Actinobacteria included *Atopobium* (20.3%) and *Bifidobacterium* (6.3%), and within Bacteroidota, *Prevotella* (4.0%) was identified. The abundance of *Solobacterium* and Erysipelotrichaceae UCG-006 has been associated with carbohydrate degradation during anaerobic fermentation for VFA production [35], whereas *Lachnospira* has been identified in HAc production from carbohydrate-rich substrates. *Atopobium* and *Bifidobacterium* are closely related to the production of HAc [36], HPro, and HBu [37]. Similarly, *Prevotella* is known to participate in carbohydrate degradation and HAc and HBu production [18]. All those genera are involved in both the hydrolysis and acidogenesis stages of AD, exhibiting high hydrolytic activity toward complex carbohydrates and contributing to even-chain VFA production. Their presence is in accordance with the VFA distribution presented in Figure 1. The higher number of genera observed at a lower pH and shorter HRT suggests that the fermenter environment became more dynamic and selective, favoring a variety of acid-tolerant, fast-growing, and metabolically flexible microorganisms. The combination of acid stress, reduced residence time, and incomplete substrate degradation likely created diverse ecological niches that supported a broader microbial community. In fact, their metabolic flexibility was supported by the similar results attained in terms of VFA bioconversion efficiencies and distribution profiles (Table 1 and Figure 1). Although microbial dissimilarity was observed among the reactors, the reproducibility of the results suggested the presence of metabolic redundancies, which conferred high process stability and robustness under dynamic conditions. Moreover, being a more diverse microbiome in which different microbial kinetics might take place could also support the similarity in VFAs for all evaluated microbiomes. The HRT decrease to 11 d in R3 prevented any genus from dominating, allowing coexistence and greater evenness in the microbial community. Additionally, with short retention and a low pH, the substrate was hydrolyzed to a greater extent (60% VS removal), which may result in a greater accumulation of a mix of intermediates (e.g., sugars, amino acids) and potentially reduce VFA production due to limited conversion. The increased hydrolysis could denote the predominant growth of hydrolytic bacteria over acidogenic ones.

## 3. Materials and Methods

### 3.1. Inoculum and Substrate

The anaerobic sludge used as the inoculum during the experimental assays was sourced from the anaerobic digester of the Wastewater Treatment Plant (WWTP) in Valladolid, Spain. The inoculum presented a VS/TS of 0.7± 0.1 and pH of 7.1± 0.2.

The carrot residues used as the substrate were collected from an agricultural company (Valladolid, Spain). The selection of this substrate was based on its high carbohydrate content (Table 3), which has great potential for VFA production. To prepare the substrate, 2.5 kg of carrots were crushed and then blended with 4.0 L of water to obtain a pulp to be fed into the reactors. The substrate was characterized (Table 3) by experimentally measuring total and soluble chemical oxygen demand (TCOD and SCOD, respectively), total and volatile solids (TS and VS, respectively), total Kjeldahl nitrogen (TKN), pH and the percentage of carbohydrates, proteins, lipids and ash.

### 3.2. Anaerobic Fermentation

To evaluate the effect of pH, three semi-continuous stirred tank reactors (CSTRs) were operated with a working volume of 1.0 L and a headspace of 0.5 L. Two of the reactors were maintained at a constant temperature of 25 °C throughout the experiment, with an organic loading rate (OLR) of 3.3 g COD/Ld and an HRT of 14 days. The experiment was conducted over three residence times to achieve stable operating conditions (approximately from day 25 to day 50 of fermentation). The operational parameters were selected based on previous studies that identified them as optimal for VFA production from carbohydrate-rich substrates [12]. These conditions were reported to promote the metabolic activity of acidogenic bacteria and maximize VFA accumulation, while minimizing the activity of methanogenic archaea. In this case, two pHs were evaluated, namely, pH 5.1 ± 0.1 (R1), as this would avoid the need for pH control while allowing the reactor to naturally acidify, and 6.0 ± 0.1 (R2), which is the pH reported to be in the optimum range for the anaerobic fermentation of carbohydrate-rich substrates [6]. Because in acid-phase fermenters a higher OLR can favour acidogenesis over methanogenesis, the pH that mediated higher bioconversion was evaluated at a higher OLR (4.1 g COD/Ld) by operating a third CSTR (R3). When using CSTRs, OLR and HRT are mathematically linked through feed rate and biologically linked through microbial kinetics. Concomitantly, with an increase in OLR, HRT decreases and this has also a direct effect on VFA productivity. This parameter is a key performance indicator in a bioprocess, whereby the efficiency of the system to convert substrates into desired products within a given time is shown. Aiming to increase VFA productivity, the increase in OLR also shortened the HRT to 11 days.

Once the AF reached a steady state, the bioprocesses were evaluated via bioconversion, acidification and hydrolysis efficiencies, which were calculated using the following equations, respectively:

(1) $$\%\;Bioconversion=\frac{{COD(VFAs)}_{Effluent}}{{TCOD}_{Influent}}\cdot 100$$

(2) $$\%\;Acidification=\frac{{COD(VFAs)}_{Effluent}}{{SCOD}_{Effluent}}\cdot 100$$

(3) $$\%\;VSremoval=\frac{{VS}_{Influent}-{VS}_{Effluent}}{{VS}_{Influent}}\cdot 100$$

where

COD (VFAs)_Effluent_: sum of the concentrations of acetic (HAc), propionic (HPro), isobutyric (isoHBu), butyric (HBu), isovaleric (isoHVal), valeric (HVal) and caproic acids (HCa) at the system outlet, expressed in terms of COD (g COD/L). Each acid was multiplied by its stoichiometric value which corresponds to 1.066, 1.512, 1.816, 1.816, 2.037, 2.037 and 2.2, respectively.

TCOD_Influent_: total COD concentration at the system inlet (g/L)

SCOD_Effluent_: soluble COD concentration at the system outlet (g/L)

VS_Influent_: volatile solids concentration at the system inlet (g/L)

VS_Effluent_: volatile solids concentration at the system outlet (g/L)

Productivity (Equation (4)) was calculated during the steady state of VFA production, as follows:

(4) $$\mathrm{VFAs}\;\mathrm{Productivity}\;(g/\mathrm{Ld})\;=\;\frac{\mathrm{SCFAs}\;(g/L)\;}{\mathrm{HRT}\;(d)}\;$$

### 3.3. Process Monitoring

To monitor the process and characterize the substrate, the following parameters were analyzed: TCOD, SCOD, TS, VS, TKN, carbohydrates, ammonium nitrogen (NH_4_^+^-N) and pH. Both COD measurements were taken using commercial kits (ISO 15705, Merck, Darmstadt, Germany). NH_4_^+^-N was determined using the kits (Spectroquant^®^ 1.000683, Merck). pH measurements, as well as TKN, TS and VS analyses, were conducted following the methodology described in APHA [38]. Carbohydrate content was analyzed using the phenol-sulfuric acid method [39]. Protein content was calculated by multiplying the TKN value by the conversion factor 6.25 [40], and lipid content (%) was calculated as the difference between 100 and the sum of the percentages of protein, carbohydrates and ash.

VFAs were analyzed using an Agilent 7820 gas chromatograph (GC, Santa Clara, CA, USA), equipped with a TEKNOCROMA Packed column with helium as the carrier gas at a flow rate of 45 mL/min. The injector temperature was maintained at 350 °C, while the oven temperature was increased to 180 °C. The FID detector was maintained at 350 °C.

The gas phase composition was analyzed using a Bruker 430 GC gas chromatograph (Billerica, MA, USA). A Porabond (Agilent) × Molesieve (Bruker) column was used, with helium as the carrier gas at a pressure of 18 psi. The injector temperature was set at 150 °C, the oven temperature at 45 °C, and the TCD detector was maintained at 200 °C.

### 3.4. Microbial Community Analysis

For microbial community analysis, samples from the inoculum and from the steady-state condition of each reactor were collected. Samples were preserved at −80 °C until DNA extraction. DNA was extracted using a FastDNA SPIN Kit for Soil (MP Biomedicals, LLC, Irvine, CA, USA). DNA concentration and quality were subsequently assessed by spectrophotometry using a Nanodrop Spectrostar Omega (BMG Labtech, Ortenberg, Germany). For the determination of bacterial and archaeal communities, the hypervariable V3–V4 regions of the 16S rRNA gene were amplified using primers 341F (CCTACGGGNGGCWGCAG) and 805R (GACTACHVGGGTATCTAATCC). The resulting amplicons were sequenced on a MiSeq platform (Illumina, San Diego, CA, USA) at FISABIO (Valencia, Spain). Bioinformatic analysis of the sequences was carried out following the procedures described by Greses et al. [28].

## 4. Conclusions

This investigation has demonstrated that operating CSTRs fed with moderately complex carbohydrate-rich feedstock at a low HRT (11 d) and high OLR (4.4 g COD/Ld) did not compromise bioconversion efficiencies or the selectivity of the VFA production profile. Using carbohydrate-rich substrates combined with a temperature of 25 °C led to a VFA bioconversion efficiency of around 45% and acidification efficiency of 84%. The shorter HRT and higher OLR had a dual effect, by which (i) a greater genera diversity was promoted and (ii) the hydrolytic activity of the microbial system was further exploited. These two effects supported a similar VFA productivity and VFA distribution profile, with a predominance of acetic and butyric acids. The significance of these results lies in the fact that higher organic loading rates enhance the space–time yield of the fermenter, enabling greater product output from the same reactor volume. These findings suggest that the common assumption that longer hydraulic retention times and lower organic loading rates enhance process stability may not directly apply when the goal is volatile fatty acid production, as more dynamic conditions can actually promote higher microbial diversity and acidogenesis. These insights open the door for future studies to refine dynamic operation strategies that maximize targeted VFA yields and support scalable, high rate acidogenic fermentations.

## Figures and Tables

**Table 1:** Mean values measured during the steady-state phase of the three reactors.

Reactor	R1	R2	R3
**pH**	**5.1 ± 0.1**	**6.0 ± 0.1**	**5.1 ± 0.1**
**HRT (d)**	**14**	**14**	**11**
**OLR (g COD/Ld)**	**3.3**	**3.3**	**4.1**
**TCOD (g/L)**	43.3 ± 2.4	36.5 ± 2.9	37.1 ± 4.6
**SCOD/TCOD (%)**	65.4 ± 4.0	71.2 ± 5.6	63.8 ± 4.4
**TS (g/L)**	23.2 ± 0.2	26.8 ± 0.6	20.2 ± 4.6
**VS/TS (%)**	61.7 ± 1.2	59.4 ± 1.7	68.9 ± 2.2
**VFAs (g/L)**	15.9 ± 1.4	13.7 ± 2.4	11.7 ± 1.4
**VFAs (g COD/L)**	24.8 ± 2.5	21.0 ± 3.9	19.6 ± 1.9
**g VFAs/Ld**	1.13 ± 0.1	1.00 ± 0.2	1.05 ± 0.1
**Bioconversion (%)**	53.6 ± 5.4	45.4 ± 8.5	43.7 ± 4.5
**Acidification (%)**	87.7 ± 9.7	80.5 ± 13.1	83.7 ± 10.3
**VS removal (%)**	53.2 ± 1.3	47.9 ± 0.5	60.1 ± 3.2

**Table 2:** Biodiversity indexes of the inoculum and steady-state reactors.

Sample	Observed OTUs	Shannon Index
Inoculum	477	4.09
R1-pH 5- HRT 14 d- OLR 3.3 g COD/Ld	53	1.17
R2-pH 6- HRT 14 d- OLR 3.3 g COD/Ld	53	0.34
R3-pH 5- HRT 11 d- OLR 4.1 g COD/Ld	103	2.37

**Table 3:** Characterization of the substrate used in anaerobic fermentation (mean ± standard deviation).

	Carrot Residue Pulp
SCOD/TCOD	0.6 ± 0.1
TS (g/L)	42.7 ± 0.3
VS/TS	0.8 ± 0.1
TKN (g N/L)	0.4 ± 0.0
Carbohydrates (*w*/*w* %)	66.1 ± 0.0
Proteins (*w*/*w* %)	7.4 ± 1.4
Lipids (*w*/*w* %)	16.3 ± 4.0
Ash (*w*/*w* %)	10.0 ± 2.6
pH (25 °C)	5.8 ± 0.2

## Data Availability

Data will be made available upon request.
